# Smart-Phone Based Magnetic Levitation for Measuring Densities

**DOI:** 10.1371/journal.pone.0134400

**Published:** 2015-08-26

**Authors:** Stephanie Knowlton, Chu Hsiang Yu, Nupur Jain, Ionita Calin Ghiran, Savas Tasoglu

**Affiliations:** 1 Department of Biomedical Engineering, University of Connecticut, Storrs, CT, 06269, United States of America; 2 Department of Mechanical Engineering, University of Connecticut, Storrs, CT, 06269, United States of America; 3 Department of Computer Science and Engineering, University of Connecticut, Storrs, CT, 06269, United States of America; 4 Department of Medicine, Beth Israel Deaconess Medical Center, Harvard Medical School, Boston, MA, 02115, United States of America; Brandeis University, UNITED STATES

## Abstract

Magnetic levitation, which uses a magnetic field to suspend objects in a fluid, is a powerful and versatile technology. We develop a compact magnetic levitation platform compatible with a smart-phone to separate micro-objects and estimate the density of the sample based on its levitation height. A 3D printed attachment is mechanically installed over the existing camera unit of a smart-phone. Micro-objects, which may be either spherical or irregular in shape, are suspended in a paramagnetic medium and loaded in a microcapillary tube which is then inserted between two permanent magnets. The micro-objects are levitated and confined in the microcapillary at an equilibrium height dependent on their volumetric mass densities (causing a buoyancy force toward the edge of the microcapillary) and magnetic susceptibilities (causing a magnetic force toward the center of the microcapillary) relative to the suspending medium. The smart-phone camera captures magnified images of the levitating micro-objects through an additional lens positioned between the sample and the camera lens cover. A custom-developed Android application then analyzes these images to determine the levitation height and estimate the density. Using this platform, we were able to separate microspheres with varying densities and calibrate their levitation heights to known densities to develop a technique for precise and accurate density estimation. We have also characterized the magnetic field, the optical imaging capabilities, and the thermal state over time of this platform.

## Introduction

Magnetic manipulation is a broadly applicable method of suspending and controlling orientation and position of objects [1–10]. One promising extension of this field is suspending and levitating objects in paramagnetic solution within a magnetic field. This technique has been widely applied to separate objects based on their density and magnetic susceptibility relative to the suspending medium [11–14]. This ability has proven useful for separating foods, including determination of the fat content in milk, cheese, and peanut butter and comparison of a variety of grains on the basis density [15]. It has been applied to characterization of trace particles of forensic evidence [16], crystal polymorphs [17], and polymer composition [18]. Density-based separation via magnetic levitation has been used extensively to monitor chemical reactions over time [18], measure the degree of protein binding [19–21] and, more recently, perform quantitative immunoassays via binding of antigens and antibodies and observing the change in density upon binding [22]. Magnetic levitation has been shown to be useful for guiding self-assembly of objects [23–25] as well as noncontact orientation of objects [26]. Though magnetic levitation is a well explored method, its potential applications can be expanded by using smart-phone attachable portable platforms with smaller magnets for levitation of micron-scale objects.

Density quantification can quickly and accurately analyze micron-scale materials for material characterization and quality assessment [27]. As magnetic levitation depends on the relative density between the levitated object and the suspending medium, the levitation height of a suspended object can be used to characterize these properties of either the levitating objects or the suspending medium. Therefore, the density of micro-objects may be quantified using this platform by directly correlating the average levitation height with the average density and the deviation of levitation height as the variability in bead density. The use of density as a characterization tool via a lightweight, inexpensive, smart-phone attachable platform may be broadly applied to many fields, including but not limited to characterization of food products on-site in food processing units, diagnosis of medical diseases and conditions, and detection of minute differences in the densities of micron-scale objects or small amounts of liquids for quality control.

Earlier magnetic levitation-based studies were performed using large setups and strong magnets, each measuring 5 by 5 by 2.5 cm and quantification of density was done based on the levitation height as measured with a ruler [15–21]. More recently, a small-scale magnetic levitation platform has been presented with the magnetic field created by magnets 2 by 5 by 50 mm in size [13], which is 125 times smaller in volume compared to magnets used previously [15–22]. Levitation on a micron scale reduces both the attachment size and the risk of the setup interfering with surrounding electronics, minimizes the quantity of reagents needed (and therefore the cost of each test), decreases equilibration time, and introduces the ability to levitate and monitor micron-sized particles. However, imaging with this device requires a microscope, which limits its portability.

Here, we propose a compact, portable, and user-friendly platform to levitate micro-objects within a magnetic field and analyze their distribution digitally and automatically. A smart-phone attachment levitates solid objects, regardless of shape or volume, which are suspended in a paramagnetic solution within a 0.98 mm by 0.98 mm square microcapillary tube. The weight of the setup is approximately 62 grams, adding to the high portability of the device. The platform is fully compatible with a Samsung Galaxy S4 smartphone running Android operating system, can be easily adapted to other smart-phones. The built-in camera of the smart-phone captures images of the sample through a secondary lens. A custom-designed algorithm-based Android application automatically analyses the distribution of particles with respect to the known field of view. This platform does not require an internet connection and achieves label-free and sensitive density measurements of solid objects. Process automation streamlines the analysis and reduces user variability and subjectivity, ultimately maximizing the accuracy of density measurements.

## Methods

### Design and Fabrication of the Platform Via 3D Printer

**Fig 1:**
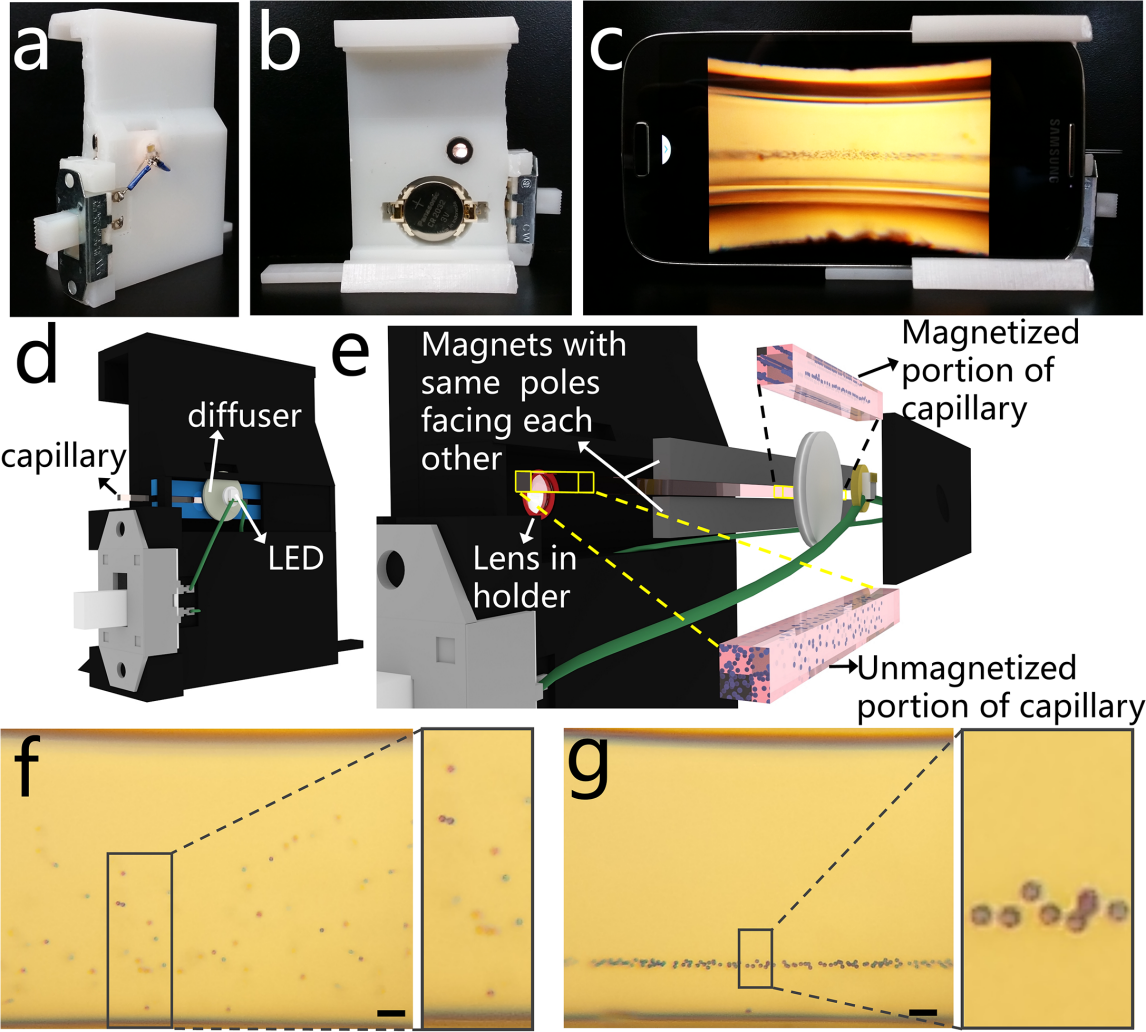
A smart-phone attachable lightweight platform to separate micro-objects based on their densities. **(a-b)** Front and back views of the 3D printed magnetic levitation platform. **(c)** Imaging of levitating microspheres using the smartphone camera and the 3D printed attachment. **(d)** 3D schematic diagram of the smart-phone attachment, where a disposable microcapillary filled with the sample is inserted from the side for imaging. **(e)** Magnified illustration of the platform showing the LED illumination, a ground glass diffuser, two rare-earth permanent magnets with the same poles facing each other, and 3D printed lens holder. Microspheres in the magnetic field are levitated and confined and those outside of the magnetic field are distributed randomly. **(f-g)** Captured image of 10 μm polystyrene microspheres (scale bar is 100 μm), **(f)** at t = 0 and **(g)** levitated and confined at equilibrium.

The smartphone density analysis platform (Fig 1A–1C, S1 Fig) consists of a 3D-printed smart phone attachment custom designed to attach to a Samsung Galaxy S4 (136.6 mm length by 69.8 mm width by 7.9 mm depth). It was designed using TinkerCAD and printed on a Form 1+ high-resolution stereolithography 3D printer with white photoreactive resin (Formlabs, Somerville, MA). The platform includes a magnetic field into which a sample is loaded, an optical lens which magnifies an illuminated image of the sample and projects it to the built-in camera in the smart-phone, and a custom-developed Android application to analyze the image and quantify the levitation height of the sample (Fig 1D and 1E). By the magnetic field generated by two magnets, randomly distributed microparticles inside microcapillary move toward an equilibrium line and form a horizontally aligned cluster (Fig 1F and 1G). The levitation location of a particle in the magnetic field depends on an equilibrium between buoyancy forces (due to relative volumetric mass density between the object and the suspending medium) and magnetic forces (due to the relative magnetic susceptibility between the object and the paramagnetic suspending medium). The paramagnetic medium used here is gadobutrol, a gadolinium chelate (Gadavist), which is FDA approved for injection as an MRI contrast agent, diluted in Hank’s Balanced Salt Solution, HBSS, which maintains the pH and osmotic balance of the medium. Thus, the procedures presented here are fully compatible with biological materials, offering the ability to quantify the density of samples such as blood and blood components.

### Optical Design and Imaging Procedure

The optical component of the attachment includes a battery-driven LED to illuminate the sample (Fig 1D and 1E). The LED is connected in series to a CR 2032 3V battery (CR2032, Panasonic, Newark, NJ) and a slide switch (GF-1123-0025, CW Industries, Southampton, PA) for user operation. From the LED, the light travels through a 1500 grit ground glass diffuser (DG05-1500, Thor Labs, Newton, NJ), selected to provide uniform illumination to the sample across the entire field of view. A 4-f imaging system is implemented to magnify the image of the sample and project it onto the built-in smart-phone camera (Sony IMX135, 1.12μm pixel pitch, 4208x3120 pixels). The image is magnified by an external aspheric lens (87–161, Edmund Optics, Barrington, NJ) with an outer diameter of 6.33 mm, effective focal length of 4.03 mm, and numerical aperture of 0.64. This lens is fixed into the 3D printed attached and aligned with the built in camera lens (which has an effective focal length of 4.2 mm) such that the focusing distance between the two lenses and between the lens and the sample is optimized experimentally and fixed in the 3D printed attachment design. Fine focusing is effectively achieved by the autofocus capabilities of the built-in camera application on the Samsung Galaxy S4. The field of view captures the entire height of the magnetic field at a 2X digital magnification, including the 0.7 mm range of levitation for the sample in the microcapillary tube and can image microspheres when they are distributed homogeneously upon entering the magnetic field (Fig 1F) and as they approach equilibrium (Fig 1G).

### Characterization of Imaging Capabilities

An image of the levitating micro-objects is magnified by the secondary lens and captured using the built-in camera of the smartphone. The useful field of view (and the area in which the application will analyze the levitation height) has been characterized in terms of the microsphere sharpness, image distortion, and uniformity of illumination as a function of horizontal position within the acquired field of view. Vertical image distortion was determined by measuring the distance between the magnets in 10-pixel-wide vertical cross-sections iteratively across the field of view. The percent increase in the field of view height was calculated for each horizontal cross-section as the percent difference between the measurement taken in the cross-section and the same measurement taken at the centerline. Uniformity was calculated by taking the average of the pixel intensities in 10-pixel-wide vertical cross-sections of the area between the magnets iteratively across the field of view. Sharpness was determined as the maximum change in intensity between 2 adjacent pixels on the edge of a microsphere along a line drawn radially from the center of the microsphere outward. The sharpness of each microsphere was plotted as the steepest pixel gradient versus the horizontal distance from the center of the steepest gradient.

### Thermal Quantification of the Magnetic Levitation Platform

To evaluate the potential effects of thermal flux from the smart-phone due to running the smart-phone application and LED, several representative locations were identified and marked at the back surface of the smart-phone. The temperatures at these locations were measured using a thermocouple temperature meter (B&K Precision) as a function of time while the LED was on, the screen was on, and the application was running. Based on these results, the camera was identified as the most critical component in terms of heat accumulation. The cooling times required between separate trials were therefore determined based on the camera’s cooling time. The effect of thermal release from the camera on the sample in the microcapillary was quantified with a room temperature 50 mM gadolinium solution. At time 0, the application was opened, the LED was turned on, and the capillary tube was placed between the magnets. The capillary tube was kept inside the holder for different time periods: 2, 5, 10, 20, 30, 40, 50, 60, and 120 minutes and after each elapsed time, the tube was removed and the temperature of capillary tube and the gadolinium solution inside the tube were measured immediately. This experiment was repeated 6 times for each elapsed time period with an optimized time to cool to the original recorded temperature (25.6 ±1°C) between experiments.

### Smart-Phone Application

This platform is supported by a custom-developed Android application running on the same smart-phone. The Android application was developed in Android Studio, which is an Android Developer Tool (The Android Open Source Project, developer.android.com). The custom-developed application processes the array of pixel intensities, where the x-coordinate corresponds to the horizontal position and the y-coordinate corresponds to the vertical height. The algorithm performs two main steps:

1. Scanning the pixels in each row, averaging the intensities of pixels, and storing them in an array indexed by the y-direction height of the row in pixels.
2. Scanning the pixels in each row, finding the gradients of pixel the intensity changes in the x-direction, and saving them in an array indexed by the y-direction height of the row in pixels.

The first array values generally peak at three locations: **(region 1)** the region starting from the upper edge of the microcapillary to the top edge of the image, **(region 2)** the region starting from the lower edge of the microcapillary to the bottom edge of the image, and **(region 3)** the confinement region where microspheres are located. The second array values mainly peak at again three locations: **(region 1)** the top and **(region 2)** the bottom edges of the capillary where they meet with the magnets and **(region 3)** as in the first array, the confinement region where microspheres are equilibrated. Based on these two arrays, region 3 is identified. A Gaussian distribution is fitted to the pixel intensity data and its mean and standard deviation (σ) are calculated in pixels. Based on the standard deviation, the width of bead confinement (~4σ) is estimated.

The magnet boundaries are located by iteratively comparing the gradients of pixel intensities in the y-direction of one pixel with that of the neighboring five pixels. The magnet boundary towards the top of the image (henceforth referred to as the ‘top magnet’) serves as the reference point for calculating the levitation height of the particles of interest. The same technique is used to identify the edges of the microcapillary as is used to identify the magnet bounds. The gradient of pixel intensity of the first pixel near the levitating objects is successively compared with the neighboring pixels towards the top until the top magnet is encountered or the gradient difference exceeds 20% to locate the upper edge of the microcapillary. Similarly, the lower edge is located. The distance between the inner edges of the microcapillary from top to bottom is known to be 0.7 mm. Based on this constant value and the number of pixels between the edges of the microcapillary, a conversion factor for pixels to mm is obtained. This is then used to convert the levitation height and standard deviation from pixels to mm and these values are displayed in the user interface in millimeters.

### Magnetic Levitation of Micro-Objects

10 μm Polybead polystyrene blue dyed microspheres (Polysciences, Inc., Warrington, PA 18976) were used to test the time-dependent magnetic levitation and the effect of the gadolinium concentration on levitation height. To correlate density to levitation height, eight density standard microspheres were obtained: 0.96, 0.98, 0.995, 1.13, 1.025, 1.050, 1.070, and 1.090 g/cc (Cospheric, Santa Barbara, CA 93111). To perform the magnetic levitation tests described here, microspheres are suspended in a Gadolinium-based (Gd^+^) paramagnetic medium, Gadavist (Bayer, Whippany NJ 07981) diluted to the desired concentration in Hank’s Balanced Salt Solution (55021C, Sigma, St. Louis, MI). Objects of interest (microspheres) suspended in solution are drawn into a 0.98 mm by 0.98 mm square glass disposable microcapillary with 0.14 mm walls (8270–50, Vitrocom, Mountain Glass, NJ) by the capillary effect. The ends of the capillary tube are sealed with Critoseal to prevent leakage (215003, Leica Biosystems St. Louis, LLC, St. Louis, MO). Then, the sample is slid into the 1 mm space between magnets.

To quantify the microsphere focusing over time, the upper and lower limits of the region in which the microspheres were confined at periodic time points was measured and reported in S1 Fig. Nine different concentrations of gadolinium paramagnetic solution were tested between 20 mM gadolinium in (a) to 250 mM in (i). Microspheres were added at 2.5% to each solution and levitated as described previously. Images were taken every 15 seconds with time 0 corresponding to insertion of the sample into the magnetic field until the microspheres appear to reach equilibrium (no significant vertical movement of microspheres). Each data point is an average over six trials. The difference between the top and bottom limits at each time point, or the width of confinement, is calculated and shown in S2 Fig. An exponential decay regression was fit to the confinement width data to quantify the time dependence of microsphere levitation and confinement. All regression equations had R-squared values greater than 0.94. This regression was used to determine the equilibrium time as the time required for the confinement width to reach 1.5 times the height of a single bead (10 μm), or 0.015 mm. The height at equilibrium was then calculated as the average between the upper and lower limits of confinement at the time point immediately following the equilibrium time.

To quantify the effect of particle size on equilibration time, three different sizes of polystyrene microspheres were levitated: 5.35, 10.4, and 20.0 μm in diameter. Microspheres were suspended in 50mM gadolinium solution and levitated in the apparatus as described previously. Images were taken starting when the microcapillary was inserted into the magnetic field (t = 0) and periodically until the microspheres reached equilibrium and the equilibration time was calculated as described previously.

To correlate micro-object density to levitation height, the eight density standard microspheres ranging from 0.96 to 1.090 g/cc were levitated as described previously. The equilibrium levitation height was quantified as the average levitation height of 60 microspheres taken over 6 trials relative to the bottom magnet. A linear regression line was use to calibrate the experimental equilibrium levitation height to the known microsphere density. Calibration curves were determined in five concentrations of gadolinium solution: 12.5, 25, 50, 100, and 200 mM gadolinium.

## Results and Discussion

### Characterization of Magnetic Levitation

**Fig 2:**
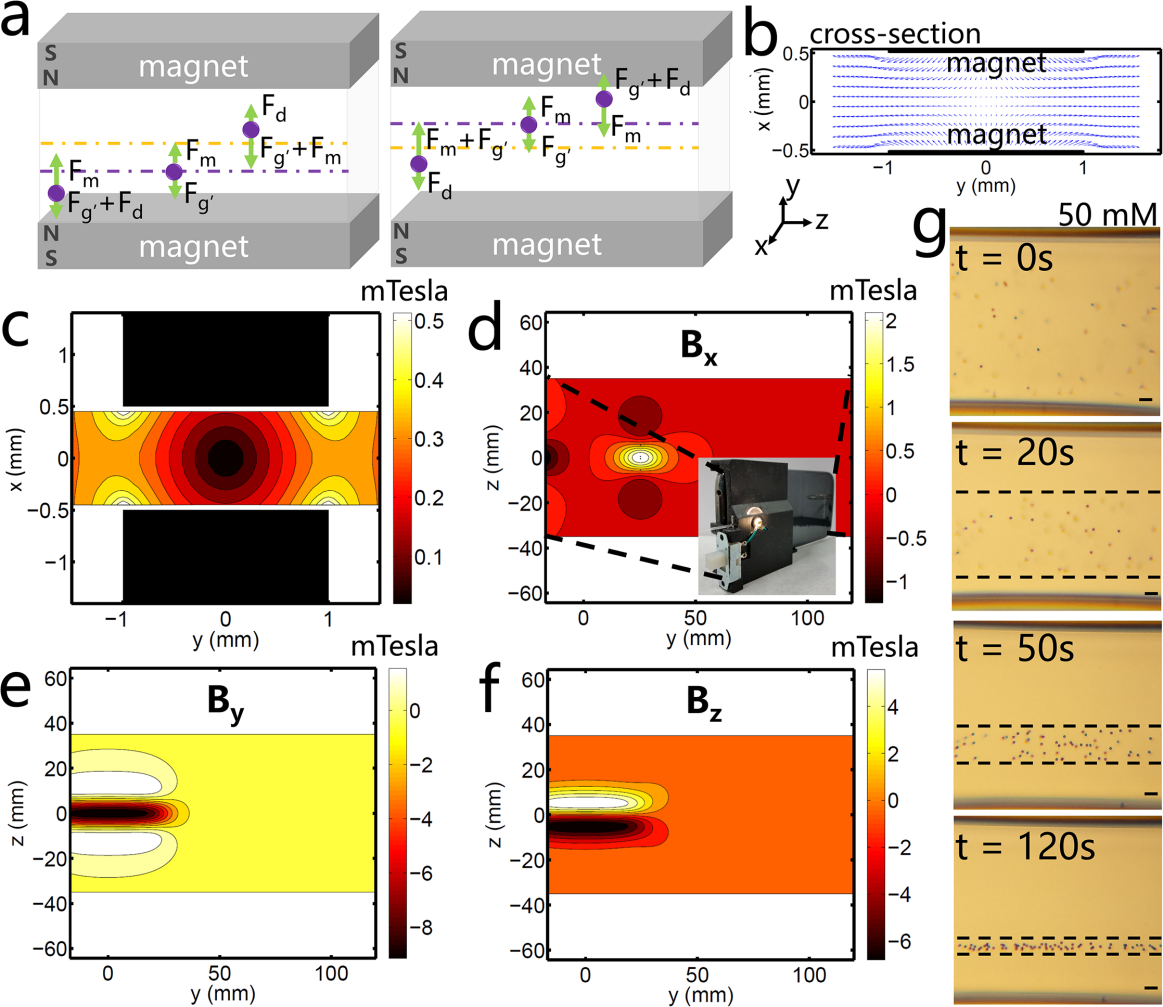
Theoretical demonstration of density-based magnetic levitation. **(a)** Representative microspheres are suspended in a paramagnetic medium for two cases: when the microspheres are more dense than the suspending medium (left) and when the microspheres are less dense than the suspending medium (right). The magnetic force (F_m_), buoyancy or corrected gravitational force (F_g’_), and drag force (F_d_) acts on microspheres, causing them to approach equilibrium (purple dotted line). F_m_ exerts a force on the microspheres in the direction of the centerline between the two magnets (orange dotted line) and changes in magnitude depending on the microsphere’s location in the magnetic field. F_g’_ exerts either a downward force (in the case of microspheres which are denser than the suspending medium, left) or an upward force (in the case of microspheres which are less dense than the suspending medium, right). F_d_ acts on the object in a direction opposite the direction of motion until the bead reaches a line of equilibrium below the centerline (as in the case of denser objects, left) or above the centerline (as in the case of less dense objects, right). At equilibrium, F_m_ and F_g’_ are equal and opposite and F_d_ has zero magnitude. **(b)** The magnetic field in the cross-section between the magnets demonstrated by the magnetic forces exerted on an object in the field; the forces have directionality toward the centerline between the two magnets and magnitude greatest near the magnets’ surfaces and approaching zero at the centerline. **(c)** Contour plot of magnitude of the magnetic field strength in the cross-sectional area at z = 0, the center between the two magnets. The magnitude of the magnetic field is constrained between 0 T and 0.4 T. **(d-f)** Contour plots showing the magnitude of the magnetic field at the back surface of the smart-phone in the **(d)** x-direction, **(e)** y-direction, and **(f)** z-direction. **(g)** Representative images of polymer microspheres in a 50 mM gadolinium solution levitating and focusing to an equilibrium height over 120 seconds.

The magnetic levitation component includes two 2 mm by 5 mm by 50 mm NdFeB permanent magnets which are magnetized through the 5 mm thickness. The magnets are fixed 1 mm apart with their magnetic axes aligned vertically and with the same poles facing each other. Mathematical modeling of the magnetic field distribution is described in S1 File, including the underlying mechanisms for levitation of microspheres in the microcapillary and the theoretical dependence of levitation height and time on several parameters. This configuration creates a magnetic field which is strongest near the magnets and approaches zero at the midline between them (Fig 2B and 2C). The sample is filled into a 0.98 mm by 0.98 mm square glass microcapillary tube by capillary action and inserted between the magnets by the user. The combined magnetic field strength generated by the permanent magnets at the back surface of the smart-phone is 8 mT (Fig 2D–2F), which does not cause any noticeable interference with the proper function of the smart-phone.

A buoyancy force acts on the microspheres due to the density difference between the microspheres and the suspending medium (Fig 2A). For example, an object which is denser than the suspending medium experiences a downward buoyancy force. Likewise, an object which is less dense than the suspending medium experiences an upward buoyancy force (Fig 2B). The microspheres simultaneously experience a magnetic force acting in the direction of the centerline between the two magnets (Fig 2A) due to the difference in magnetic susceptibility between the paramagnetic suspending solution and the microspheres. and the object approaches equilibrium at a location in the magnetic field (“height” relative to the bottom magnet) at which the magnetic force is equal and opposite to the buoyancy force. Due to drag forces, the equilibration of an object is time-dependent and equilibration time depends on the magnitude of the density and buoyancy forces (Fig 2G).

### Characterization of Imaging Capabilities

**Fig 3:**
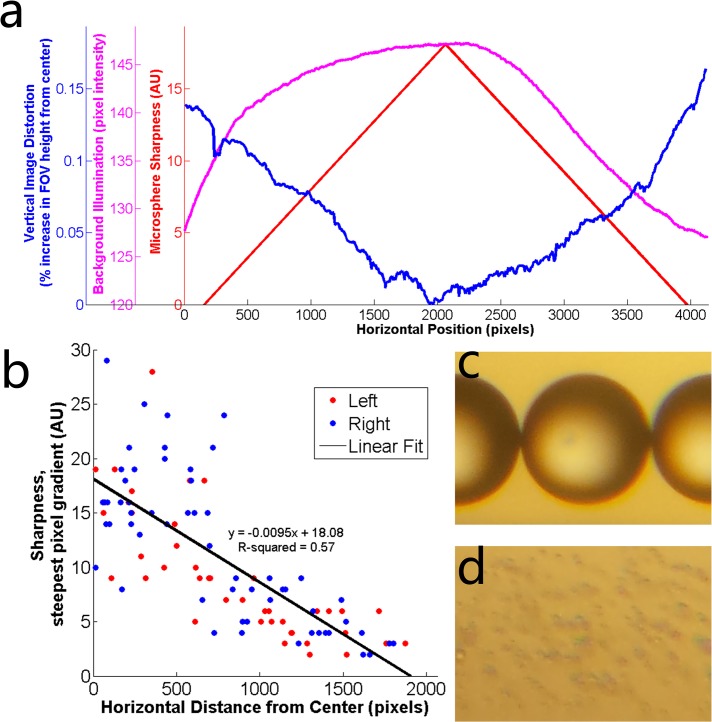
Optical quantification of density-based magnetic levitation. **(a)** Quantification of image distortion (blue), background illumination (magenta), and microsphere sharpness (red) along the horizontal field of view. Microsphere sharpness is shown as the line of best fit for the data shown in (b). **(b)** Data points representing sharpness of microspheres located at different distances from the center of the field of view. The line of best fit approximates the decrease in image sharpness as the distance from the centerline increases. Red data points represent microspheres located to the left of the centerline and blue data points represent microspheres located to the right of the centerline. **(c)** 210 μm and **(d)** 5.25 μm diameter microspheres demonstrating qualitatively the optical resolution of the platform.

Sharpness was measured for 100 microspheres across the field of view and plotted as a function of their distance from the center (Fig 3B). Linear regression analysis shows an approximately linear correlation with sharpness decreasing as the distance from the center increases. The linear approximation for sharpness as well as the vertical image distortions and background illumination are plotted as a function of the horizontal position in the field of view (Fig 3A). This data shows a degradation of image quality as quantified as in increase in vertical image distortion and a decrease in background illumination and sharpness toward the edge of the field of view. The resolution of the optical setup is demonstrated qualitatively by images of 210 μm and 5.35 μm microspheres (Fig 3C and 3D, respectively), showing degradation in resolution when magnifying the image to view the smaller microspheres. However, the platform is still able to detect micro-objects of this size and determine the levitation height of a levitated and confined group of these microspheres.

### Thermal Quantification of the Magnetic Levitation Platform

**Fig 4:**
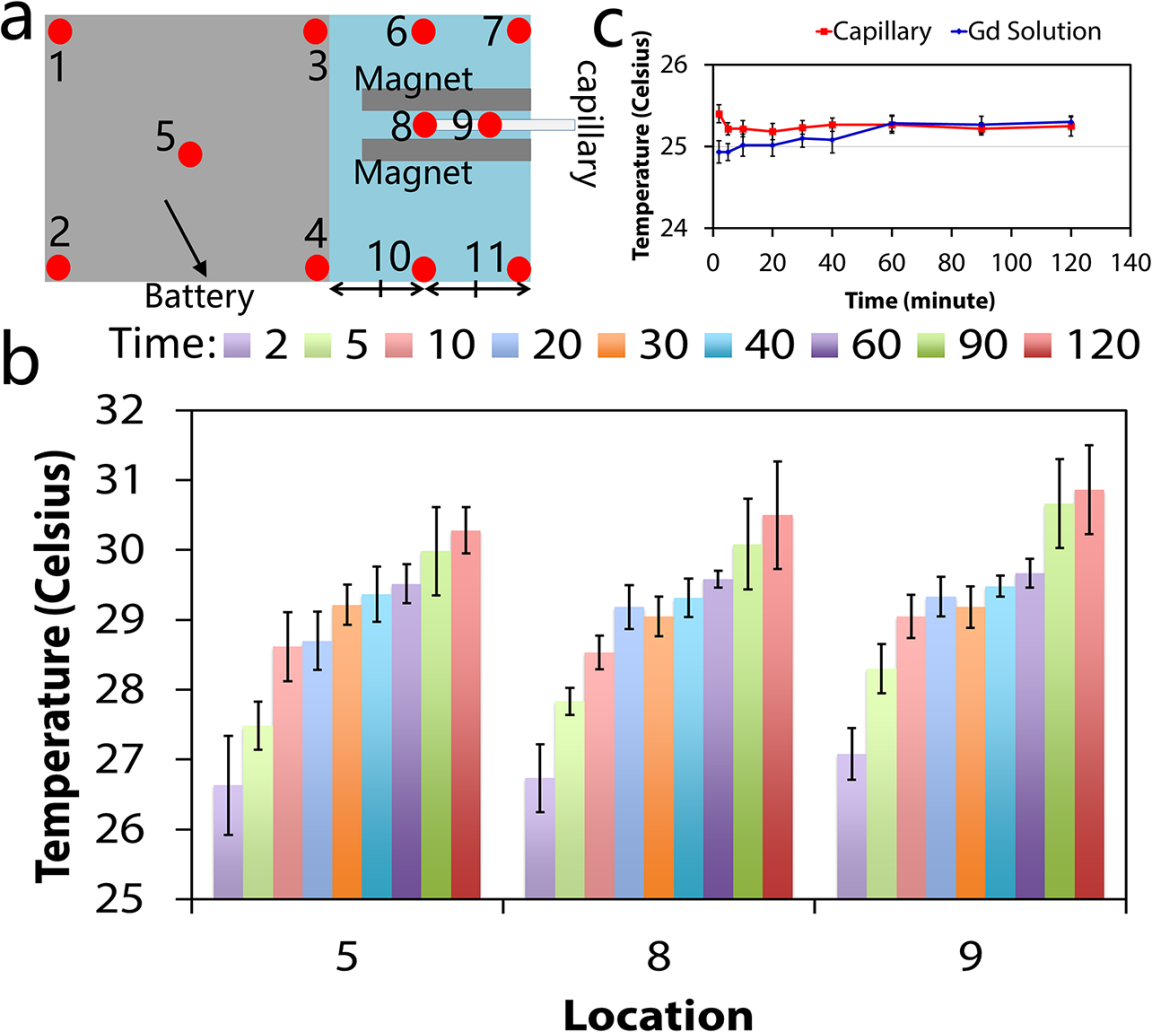
Thermal quantification of the magnetic levitation platform. **(a)** Temperatures at several locations at the back surface of the smart phone were measured while the smart-phone density estimation application was running. Location 8 corresponds to the back surface of the phone closest to the end of the microcapillary. Location 9 corresponds to the surface of the smart-phone camera. Full thermal plots are given in S4 Fig **(b)** Temperature readings over 120 minutes at locations 5, 8, and 9 at running times = 2, 5, 10, 20, 30, 40, 60, 90, and 120 minutes (n = 6). **(c)** Temperature in the gadolinium solution and capillary as a function of running time with standard deviation (n = 6).

Due to heat generation from the smart-phone and the LED during the assay, it is possible that excess heat reaches the sample in the microcapillary. This may affect the density estimation results, as an increase in temperature may cause a decrease in the density of the medium and affect the sample levitation. Therefore, the thermal characteristics of the magnetic levitation platform were quantified experimentally. Thermal quantification at the back of the smart-phone indicate that all temperatures increased from room temperature (25.6 ±1°C) to a temperature between 29°C and 31°C after 2 hours (Fig 4B; complete data set shown in S4 Fig). Thermal quantification of the gadolinium sample and the microcapillary indicate that thermal release from smart phone did not have a significant effect on the temperature of the sample and the microcapillary, which can be partly attributed to the shell of air between the capillary and the attachment and its potential insulation role (the thermal conductivity of air/resin is ~10^−1^). This implies that thermal fluctuations in the sample due to the use of the smart-phone will not significantly alter the temperature or interfere with the density determination.

### Smart-Phone Application

**Fig 5:**
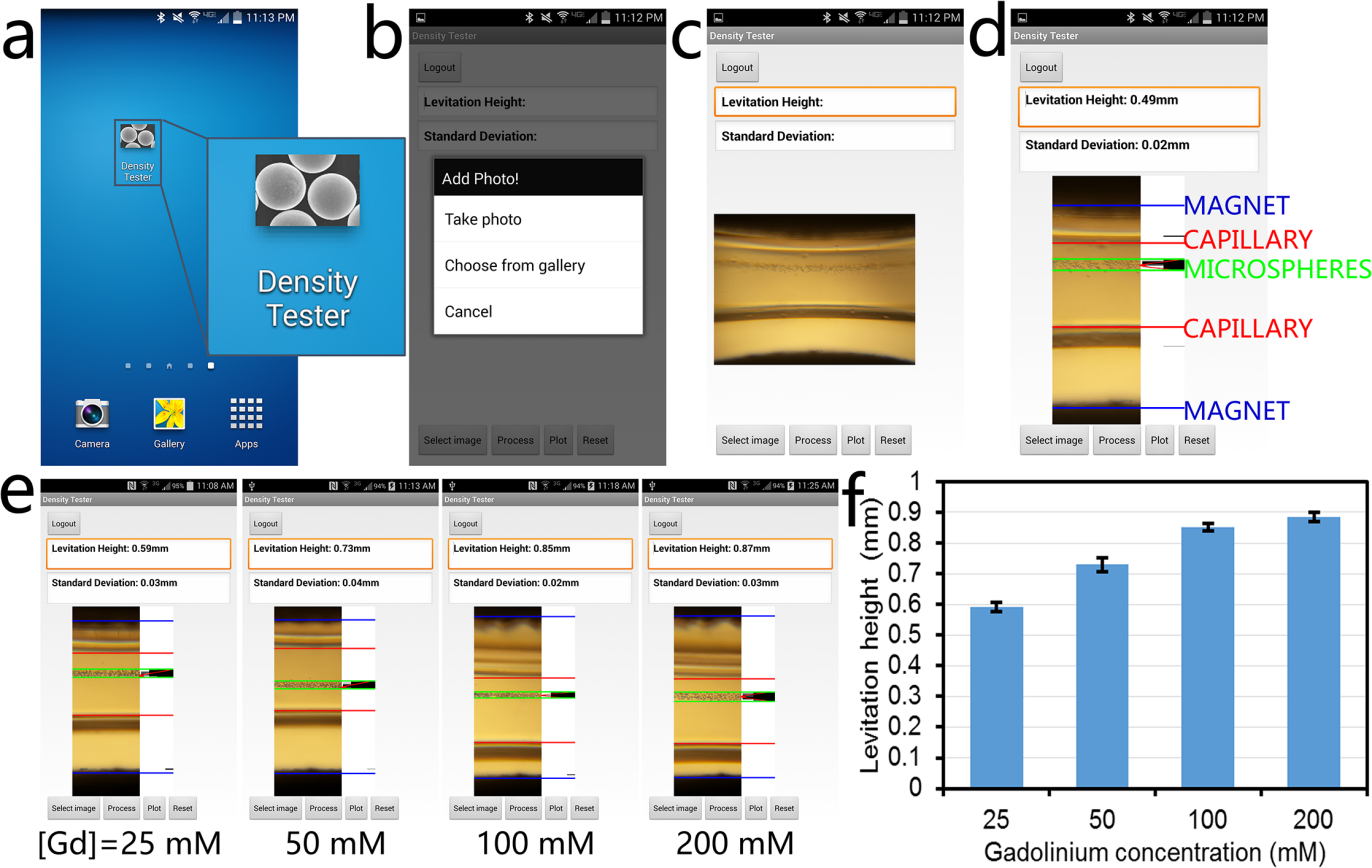
Android-based application running on the same smart phone. **(a)** Density Tester application icon. **(b)** In the smart-phone application, the user is given the option to take an image using the camera application or to choose an existing image from the gallery. The selection of ‘Choose from gallery’ enables access and processing of the images stored in the smart-phone or memory card installed on the smart-phone. ‘Take photo’ directs the user to the camera application. **(c)** Once an image is taken or chosen from memory, it is loaded into the application. **(d)** When the user selects ‘Process’, the application performs an analysis to crop the image to the useful field of view and identify the magnet edges (blue), capillary edges (red), and microsphere confinement area (green). It also calculates the Levitation Height and Standard Deviation of the microsphere confinement area as the distance from the bottom magnet (shown as the top magnet in these images) and width of the microsphere distribution, respectively. **(e)** Screenshots showing the analysis results for microspheres levitated in different concentrations of gadolinium: 25 mM, 50 mM, 100 mM, and 200 mM. **(f)** Summary of the results obtained in (e), demonstrating the ability of the application to accurately and repeatably detect different levitation heights as a function of the gadolinium concentration in the paramagnetic medium, showing a positive correlation (n = 6).

The smartphone application, named Density Tester, offers a user-friendly interface for density estimation on any Android device (Fig 5A). The application prompts the user to take a photo or to choose one from the gallery (Fig 5B). The image is then retrieved and displayed on the main activity screen (Fig 3C). When the user selects ‘Process,’ the application crops the input image to the in-focus region and detects the limits of confinement, the microcapillary edges, and the magnet edges, which are marked for the user to verify proper analysis (Fig 5D). For each image analyzed, the application displays the Gaussian fit corresponding to the pixels in the region of confinement as well as the levitation height and the standard deviation at the top of the screen (Fig 5E). The application was used to plot the dependence of levitation height on the gadolinium concentration in the suspending medium to demonstrate the accuracy and reliability of the automated analysis (Fig 5F).

### Effect of Gadolinium Concentration on Equilibrium Time

**Fig 6:**
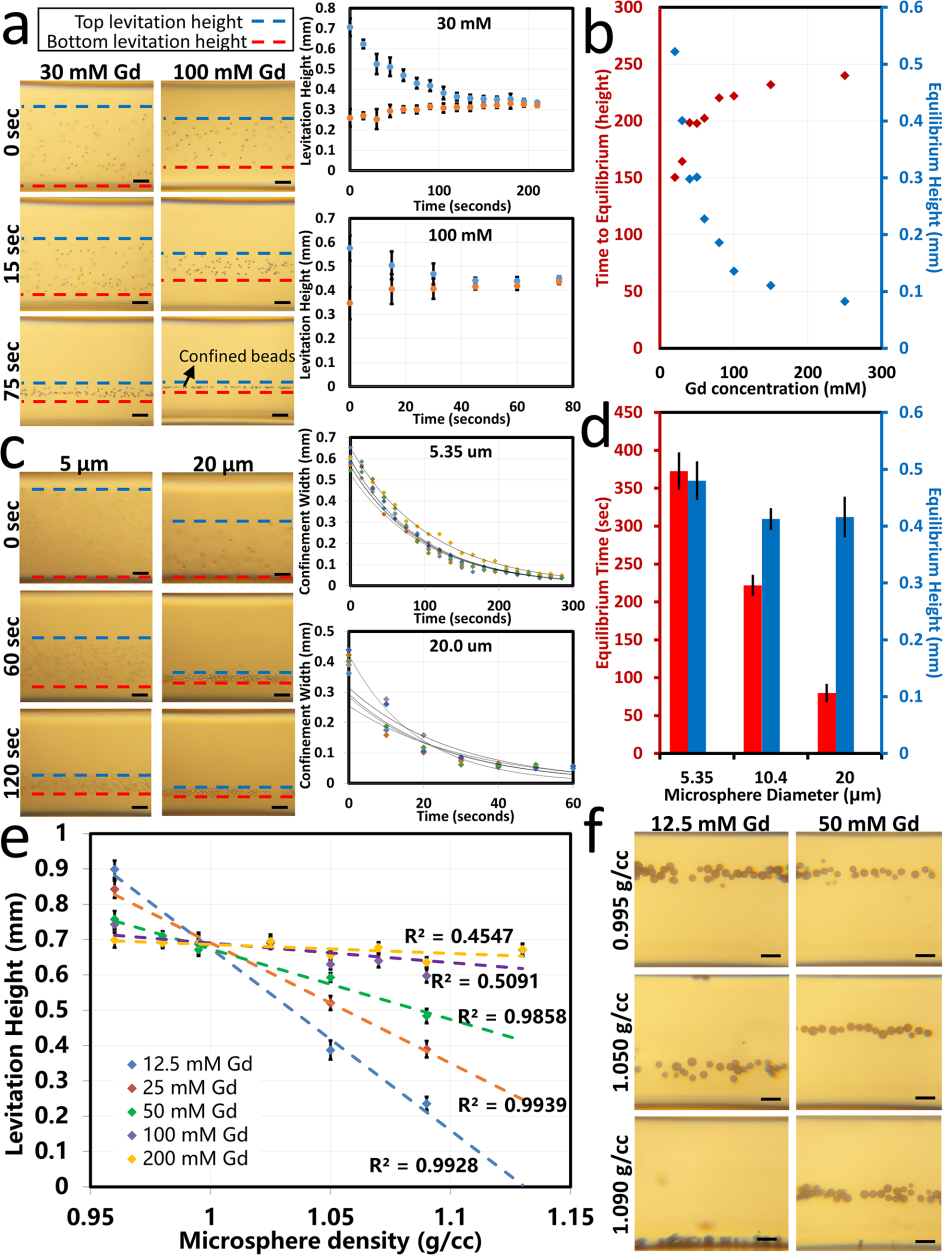
Experimental demonstration of magnetic levitation of polymer microspheres and quantification of levitation height. **(a)** Time-dependent focusing of 10 μm polystyrene microspheres in 30 mM and 100 mM gadolinium solution. Images shown at 0, 15 and 75 seconds after being placed in the magnetic field demonstrate time-dependent focusing of microspheres influenced by the gadolinium concentration. Graphs show the top (blue) and bottom (red) confinement limits of the microspheres over time in 30 mM and 100 mM gadolinium paramagnetic mediums. **(b)** Quantification of equilibrium time (red) and equilibrium height (blue) for several concentrations of gadolinium. **(c)** Time-dependent focusing of microspheres with different diameters (5.35 and 20 μm) in 50 mM gadolinium solution. Images shown at 0, 60, and 120 seconds after insertion into the magnetic field demonstrate time-dependent focusing of microspheres influenced by the microsphere size. Graphs show the width of microsphere confinement over time for 6 different trials with exponential decay approximations. **(d)** Quantification of equilibrium time (red) and equilibrium height (blue) as a function of microsphere size, demonstrating that increasing size decreases the time to equilibrium, but the size has no statistically significant effect on levitation height. **(e)** Calibration of levitation height to density using eight density standard microspheres. Five different gadolinium concentrations are used to demonstrate the ability to obtain greater resolution at lower gadolinium concentrations. **(f)** Images representing different levitation heights of three different density standard microspheres in two different concentrations of paramagnetic medium. Scale bars are 100 μm.

The levitation height of the microspheres in the magnetic field is shown to depend on the concentration of gadolinium in the suspending medium. Fig 6A demonstrates the magnetic levitation of microspheres with time lapse images. The graphs in Fig 6A show the upper and lower limits of confinement of the set of microspheres over time as they approach equilibrium (representative images shown in Fig 6A, right; full data set shown in S2 Fig). The width between the upper and lower limits of confinement over time were approximated by an exponential decay equation and used to determine the time to equilibrium as the time for the sample to reach a confinement width of 1.5 times the diameter of a single microsphere (full data set shown in S3 Fig). Fig 6B shows that there is a positive correlation between gadolinium concentration and equilibrium height. The greatest difference in equilibrium height is seen at lower concentrations of gadolinium while higher concentrations of gadolinium cause the microspheres to approach a maximum equilibrium height near the centerline between the two magnets. This is due to the fact that the microspheres tested are denser than the suspending medium, so the buoyancy force always acts in the downward direction. At the highest concentration of gadolinium, the maximum levitation height must remain below the centerline where the magnetic force is upward in order to attain equilibrium, restricting the levitation to the centerline and below. Fig 6B also shows the negative correlation between the time to equilibrium and the paramagnetic medium concentration, with more significant differences in equilibrium time observed at lower gadolinium concentrations. All cases reached equilibrium within 6 minutes within the magnetic field and in under 1 minute for the highest gadolinium concentration tested. This is due to the higher magnetic force of higher gadolinium concentrations acting on the levitating object as it moves toward equilibrium.

### Effect of Microsphere Size on Equilibration Time

Fig 6C demonstrates the focusing of microspheres with different diameters over time. The time dependent confinement of the microspheres as they approach equilibrium was quantified as the width of confinement versus time and approximated by an exponential decay equation (n = 6 data shown in Fig 6C, right). In this way, the time to equilibrium was quantified as a function of microsphere diameter between 5 and 20 μm (Fig 6D). Larger microspheres (up to 210 μm) were also tested and shown to reach equilibrium more rapidly than smaller microspheres (data not shown due to high degree of user error associated with low equilibration times).

### Density Calibration of Microspheres

The relationship between microsphere density and equilibrium levitation height was characterized using a range of eight density standard microspheres ranging from 0.96 to 1.13 g/cc suspended in five difference concentrations of gadolinium between 12.5 and 200 mM (Fig 6E and 6F). An inverse and approximately linear relationship is seen between microsphere density and levitation height. The difference in levitation height relative to density is greatest when suspended in medium with lower concentrations of gadolinium, giving the greatest resolution in the range of densities tested. At higher concentrations of gadolinium, the microspheres levitated across a smaller vertical range and near the centerline. The trend lines intersect around a density of 1 g/cc near the centerline (Fig 6E). This point give an approximation of the density of the suspending medium. At this density, there is a weak buoyancy force due to the minimal density difference between the sample and medium; this allows the microspheres to levitate toward the low magnetic force at the centerline of the magnetic field. This data provides a calibration curve with which one may determine an unknown density given an observable levitation height and a known concentration of gadolinium in the suspending medium.

## Conclusions

We have demonstrated a digital and lightweight platform to measure densities of micro-objects, which employs permanent magnets fixed with like poles facing each other, a disposable microcapillary, optical components (aspheric lens, printed lens frame, LED, and diffuser), and a custom-developed Android application running on a smart-phone. This small-scale method has been validated with densities of 0.96–1.09 g/cm^3^. Increasing the density of the paramagnetic medium by using other solvents such as a saturated salt solution (ρ = 1.204 g/mL) or chloroform (ρ = 1.4788 g/mL) would shift this measurable range toward higher density particles with densities in the range of these mediums. Similarly, a lower density medium such as ethanol (ρ = 0.789 g/mL) or hexane (ρ = 0.659 g/mL) would allow measurement of a lower range of densities. Based on the experiment shown in Fig 6E, increasing the gadolinium concentration increases the range of detection because the stronger magnetic forces can overcome greater buoyancy forces associated with much more or less dense levitating objects. On the other hand, decreasing the concentration of gadolinium in the medium improves the resolution of detection (i.e. the ability to distinguish between smaller density differences among two levitating objects) but limits the range of detection (as higher and lower densities outside the range levitate against the top and bottom edges of the capillary). Due to the small size scale of the platform, there is an upper limit to the object which may fit in the microcapillary tube with an inner dimension of 700 μm and which may be detected with precision by the detection algorithm–we estimate this dimension to be around 210 μm. There is also a lower limit to the object size which may be imaged by the camera used here. This dimension is also particularly limited by the autofocusing feature built into the camera (as only a few smart-phone models currently available offer manual control over the focus). Another limitation on this dimension is the high focusing time for smaller particle diameters as reported in Fig 6D; particles of 5.35 μm diameter took over 6 minutes to reach equilibrium. An absolute theoretical lower limit to microsphere size exists due to Brownian motion causing very small particles to remain evenly dispersed in the medium [28].

Based on the demonstrated ability to levitate and confine micro-objects, the platform here is broadly applicable, particularly for biomedical applications. Different cell types, and cell and cells with certain diseased phenotypes, have unique magnetic signatures and volumetric mass densities [13], and thus may be distinguished from one another using this platform. This strategy does not require antibodies or microscopy knowledge for diagnosis. This platform offers numerous advantages for clinicians/physicians for investigations such as white blood cell count, detection of circulating tumor cells, as well as monocyte, leukocyte, and neutrophil cytometry, which have previously been performed using a microscopy-compatible magnetic levitation setup [13]. Additionally, immuno-assays [22] have been performed using magnetic levitation to observe density changes associated with specific analyte binding. Application of this small-scale, portable, and user-friendly platform to such biological assays would revolutionize point-of-care disease diagnostics.

## Supporting Information

S1 File Mathematical modeling of the magnetic field distribution (DOCX)

S1 Fig CAD images of the 3D printed smart-phone attachment demonstrating construction of the magnetic levitation platform. Spaces for the magnets, lens, battery in a battery holder, switch, ground glass diffuser, LED, and wires are shown. These components are added post-printing. The user inserts the Samsung Galaxy S4 in the space as shown and its position is fixed upright on its side. The user inserts the sample in a 1 mm square microcapillary tube between the magnets for analysis as shown.
(TIF)

S2 Fig Time dependent confinement of microspheres in different concentrations of gadolinium. Levitation heights of the upper (blue) and lower (orange) limits of confinement of a sample of polystyrene microspheres over time. Time 0 corresponds to when the sample was inserted into the magnetic field and data was collected until the sample reached equilibrium. Nine different gadolinium concentrations are shown in a-i. Each time point is an average over six trials with error bars representing the standard deviation.
(TIF)

S3 Fig Width of confinement of microspheres in different concentrations of gadolinium. Width between the upper and lower limits of confinement of a sample of polystyrene microspheres over time. Values are calculated as the difference between the upper and lower data points shown in S2 Fig Nine concentrations of gadolinium paramagnetic solution were tested. An exponential decay equation was used to approximate the data with R^2^ greater than 0.94 in all cases.
(TIF)

S4 Fig Smart-phone Temperature Measurement. After the smart-phone camera application was turned on, the temperature was measured at 11 locations on the back surface of smart-phone (Fig 4A) for time points: 2, 5, 10, 20, 30, 40, 60, 90, and 120 minutes **(b)**. At the battery side, the temperature was measured at 5 locations (four on the edge of battery, and one at the middle of the battery). At the camera side, temperatures of six locations were measured, including the location corresponding to the end of capillary tube (8) and the temperature on the camera (9). After each measurement, the smart-phone application was turned off for cooling until the temperature of smart-phone decrease to room temperature. Six repeats for each of eleven locations at the back surface of the smart-phone. **(c)** Cooling times required between each trial for different thermal exposure times. For instance, for 90 minute experiments durations, the smart-phone was kept off for 40 minutes prior to the start of a new trial.
(TIF)
