# Supplementary material for: Smart-Phone Based Magnetic Levitation for Measuring Densities
Source: PLoS One. 2015 Aug 26;10(8):e0134400. doi: 10.1371/journal.pone.0134400 (PMC4550410; doi:10.1371/journal.pone.0134400)
Supplement: S1 File — (DOCX) [file pone.0134400.s001.docx]

**Smart-phone Based Magnetic Levitation for Measuring Densities**

**S1 File**

Stephanie Knowlton^1^, Chu Hsiang Yu^2^, Nupur Jain^3^, Ionita Ghiran^4,*^, and Savas Tasoglu^1,2,*,¥^

^1^Department of Biomedical Engineering, University of Connecticut, Storrs, CT 06269.

^2^Department of Mechanical Engineering, University of Connecticut, Storrs, CT 06269.

^3^Department of Computer Science and Engineering, University of Connecticut, Storrs, CT 06269

^4^Department of Medicine, Beth Israel Deaconess Medical Center, Harvard Medical School, Boston, MA 02115

*These authors contributed equally to this work

^¥^Corresponding Author: Savas Tasoglu (*[savas@engr.uconn.edu](mailto:savas@engr.uconn.edu)*)

Mathematical modeling of magnetic field distribution**.**

The magnetic field, B, follows:

|  | $\mathbf{B}\left( x,y,z \right)= \frac{\mu_{0}M_{s}}{4\pi}\sum_{k=1}^{2} \left( -1 \right)^{k}\int_{y_{1}}^{y_{2}} \int_{x_{1}}^{x_{2}} \frac{\left[ \left( x-x^{'} \right)\hat{\boldsymbol{x}}+ \left( y-y^{'} \right)\hat{\boldsymbol{y}}+ \left( z-z_{k} \right)\hat{\boldsymbol{z}} \right]dx^{'}dy'}{\left[ \left( x-x^{'} \right)^{2}+ \left( y-y^{'} \right)^{2}+ \left( z-z_{k} \right)^{2} \right]^{3/2}}$ | (1) |
| --- | --- | --- |

The x-component: B_x_ follows from Eq. (1)

|  | $B_{x}\left( x,y,z \right)= \frac{\mu_{0}M_{s}}{4\pi}\sum_{k=1}^{2} \left( -1 \right)^{k}\int_{y_{1}}^{y_{2}} \int_{x_{1}}^{x_{2}} \frac{\left( x-x^{'} \right)dx^{'}dy'}{\left[ \left( x-x^{'} \right)^{2}+ \left( y-y^{'} \right)^{2}+ \left( z-z_{k} \right)^{2} \right]^{3/2}}.$ | (2) |
| --- | --- | --- |

Integration with respect to x’ gives

|  | $B_{x}\left( x,y,z \right)= \frac{\mu_{0}M_{s}}{4\pi}\sum_{k=1}^{2} \sum_{m=1}^{2} \left( -1 \right)^{k+m}\int_{y_{1}}^{y_{2}} \frac{dy'}{\left[ \left( x-x_{m} \right)^{2}+ \left( y-y^{'} \right)^{2}+ \left( z-z_{k} \right)^{2} \right]^{1/2}}.$ | (3) |
| --- | --- | --- |

The remaining y’ integration can be evaluated by making a change of variable to α = y – y’. The resulting field expression is

|  | $B_{x}\left( x,y,z \right)= \frac{\mu_{0}M_{s}}{4\pi}\sum_{k=1}^{2} \sum_{m=1}^{2} \left( -1 \right)^{k+m}\ln\left[ F(x,y,z,x_{m},y_{1},y_{2},z_{k}) \right],$ | (4) |
| --- | --- | --- |
|  | $F\left( x,y,z,x_{m},y_{1},y_{2},z_{k} \right)= \frac{\left( y-y_{1} \right)+ \left[ \left( x-x_{m} \right)^{2}+ \left( y-y_{1} \right)^{2}+ \left( z-z_{k} \right)^{2} \right]^{1/2}}{\left( y-y_{2} \right)+ \left[ \left( x-x_{m} \right)^{2}+ \left( y-y_{2} \right)^{2}+ \left( z-z_{k} \right)^{2} \right]^{1/2}}$ |  |

**The y-component:** B_y_ also follows from Eq. (1),

|  | $B_{y}\left( x,y,z \right)= \frac{\mu_{0}M_{s}}{4\pi}\sum_{k=1}^{2} \left( -1 \right)^{k}\int_{y_{1}}^{y_{2}} \int_{x_{1}}^{x_{2}} \frac{\left( y-y^{'} \right)dx^{'}dy'}{\left[ \left( x-x^{'} \right)^{2}+ \left( y-y^{'} \right)^{2}+ \left( z-z_{k} \right)^{2} \right]^{3/2}}.$ | (5) |
| --- | --- | --- |

Integration with respect to y’ gives

|  | $B_{y}\left( x,y,z \right)= \frac{\mu_{0}M_{s}}{4\pi}\sum_{k=1}^{2} \sum_{m=1}^{2} \left( -1 \right)^{k+m}\int_{x_{1}}^{x_{2}} \frac{dx'}{\left[ \left( x-x^{'} \right)^{2}+ \left( y-y_{m} \right)^{2}+ \left( z-z_{k} \right)^{2} \right]^{1/2}}$ | (6) |
| --- | --- | --- |

The remaining x’ integration is evaluated using a change of variable α = x – x’. The resulting field expression is

|  | $B_{y}\left( x,y,z \right)= \frac{\mu_{0}M_{s}}{4\pi}\sum_{k=1}^{2} \sum_{m=1}^{2} \left( -1 \right)^{k+m}\ln\left[ H(x,y,z,x_{1},x_{2},y_{m},z_{k}) \right].$ | (7) |
| --- | --- | --- |
|  | $H\left( x,y,z,x_{1},x_{2},y_{m},z_{k} \right)= \frac{\left( x-x_{1} \right)+ \left[ \left( x-x_{1} \right)^{2}+ \left( y-y_{m} \right)^{2}+ \left( z-z_{k} \right)^{2} \right]^{1/2}}{\left( x-x_{2} \right)+ \left[ \left( x-x_{2} \right)^{2}+ \left( y-y_{m} \right)^{2}+ \left( z-z_{k} \right)^{2} \right]^{1/2}}.$ |  |

**The z-component:** B_z_ is given by

|  | $B_{z}\left( x,y,z \right)= \frac{\mu_{0}M_{s}}{4\pi}\sum_{k=1}^{2} \left( -1 \right)^{k}\int_{y_{1}}^{y_{2}} \int_{x_{1}}^{x_{2}} \frac{\left( z-z_{k} \right)dx^{'}dy'}{\left[ \left( x-x^{'} \right)^{2}+ \left( y-y^{'} \right)^{2}+ \left( z-z_{k} \right)^{2} \right]^{3/2}}.$ | (8) |
| --- | --- | --- |

The x’ integration is performed using a change of variable α = x – x’

|  | $B_{z}\left( x,y,z \right)= \frac{\mu_{0}M_{s}}{4\pi}\int_{y_{1}}^{y_{2}} \int_{x-x_{1}}^{x-x_{2}} \frac{z-z_{k}}{\left[ \alpha^{2}+ \left( y-y^{'} \right)^{2}+ \left( z-z_{k} \right)^{2} \right]^{3/2}}d\alpha dy'$ | (9) |
| --- | --- | --- |
|  | $= \frac{\mu_{0}M_{s}}{4\pi}\sum_{k=1}^{2} \sum_{n=1}^{2} \left( -1 \right)^{k+n+1}\left( z-z_{k} \right)\left( x-x_{n} \right)\int_{y_{1}}^{y_{2}} \frac{dy^{'}}{\left[ \left( y-y^{'} \right)^{2}+ \left( z-z_{k} \right)^{2} \right]\sqrt{\left( y-y^{'} \right)^{2}+b^{2}}},$ |  |

where b^2^ = (x – x_n_)2 + (z – z_k_)^2^. The remaining y’ integration is performed using a change of variable γ = y – y’. This gives

|  | $B_{z}\left( x,y,z \right)= \frac{\mu_{0}M_{s}}{4\pi}\sum_{k=1}^{2} \sum_{n=1}^{2} \sum_{m=1}^{2} \left( -1 \right)^{k+n+m}\tan^{-1} \left[ \frac{\left( x-x_{n} \right)\left( y-y_{m} \right)}{\left( z-z_{k} \right)}g(x,y,z;x_{n},y_{m},z_{k}) \right],$ | (10) |
| --- | --- | --- |

Here, we solved equations 4, 7, and 10 to plot magnetic field distribution (Figure 2b-f). Cells will be driven towards regions of minimal magnetic flux density (along the symmetry line, i.e. centerline between two magnets) (Figure 2b) and can be spatially confined in 3D or 2D magnetic traps. We plotted magnetic field distribution at the back surface of cell phone (Figure 2d-f).

The underlying mechanisms for levitation of beads were extensively described [[1](#_ENREF_1),[2](#_ENREF_2)], and mathematical derivations were mainly performed in [[1](#_ENREF_1),[2](#_ENREF_2)], and also covered in several other articles [[3-5](#_ENREF_3)].

**References:**

1. Furlani EP (2001) Permanent Magnet and Electromechanical Devices: Elsevier.

2. Mirica KA, Shevkoplyas SS, Phillips ST, Gupta M, Whitesides GM (2009) Measuring Densities of Solids and Liquids Using Magnetic Levitation: Fundamentals. Journal of the American Chemical Society 131: 10049-10058.

3. Mirica KA, Ilievski F, Ellerbee AK, Shevkoplyas SS, Whitesides GM (2011) Using magnetic levitation for three dimensional self-assembly. Advanced Materials 23: 4134-4140.

4. Tasoglu S, Yu CH, Gungordu HI, Guven S, Vural T, et al. (2014) Guided and magnetic self-assembly of tunable magnetoceptive gels. Nat Commun 5.

5. Tasoglu S, Khoory J, Tekin HC, Thomas C, Ghiran IC, et al. (2015) Levitational image cytometry with temporal resolution. Advanced Materials in press.
