# Supplementary figures and images for: Smart-Phone Based Magnetic Levitation for Measuring Densities
Source: PLoS One. 2015 Aug 26;10(8):e0134400. doi: 10.1371/journal.pone.0134400 (PMC4550410; doi:10.1371/journal.pone.0134400)

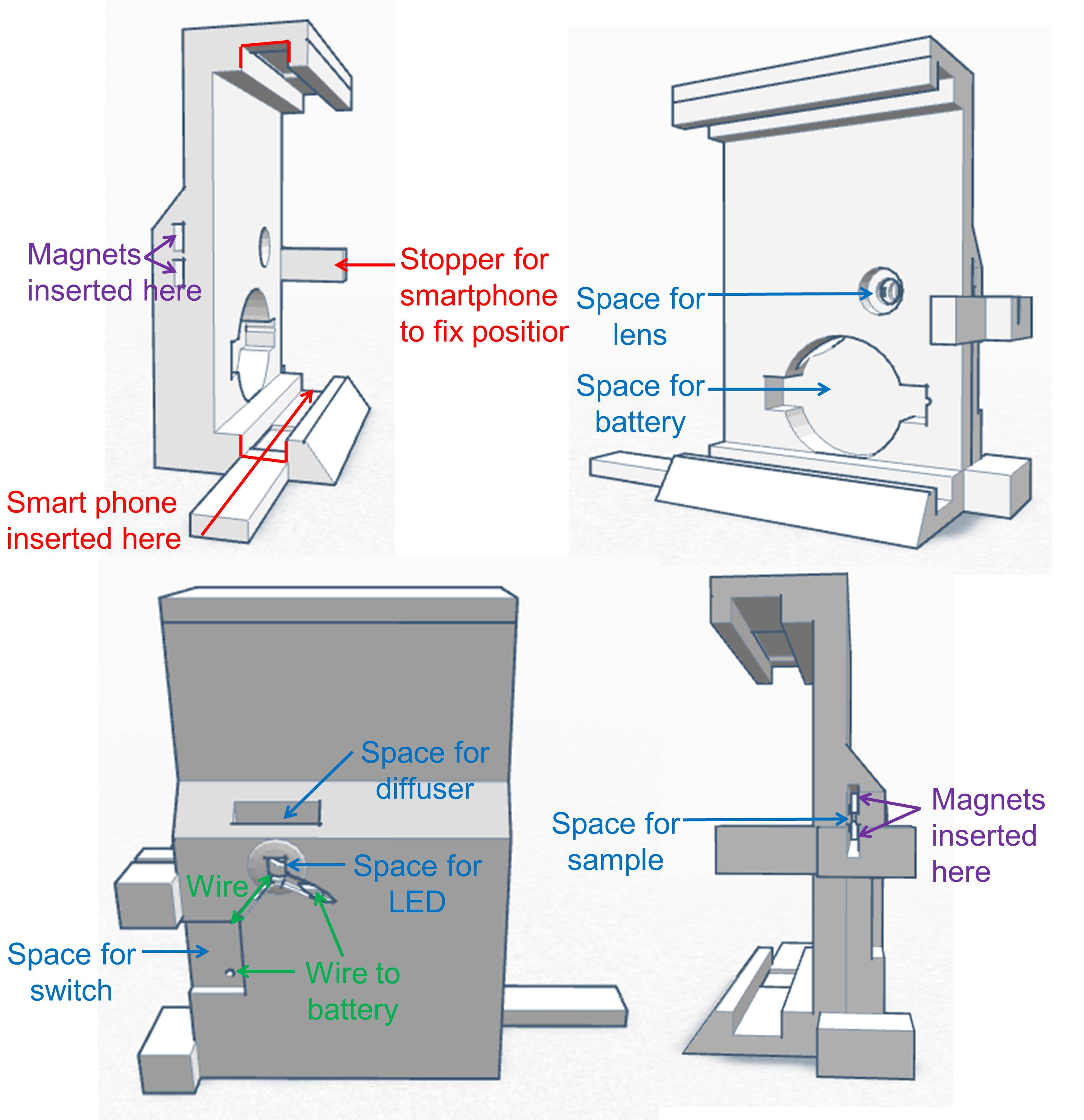

Supplement: S1 Fig — Spaces for the magnets, lens, battery in a battery holder, switch, ground glass diffuser, LED, and wires are shown. These components are added post-printing. The user inserts the Samsung Galaxy S4 in the space as shown and its position is fixed upright on its side. The user inserts the sample in a 1 mm square microcapillary tube between the magnets for analysis as shown. (TIF) [file pone.0134400.s002.tif]

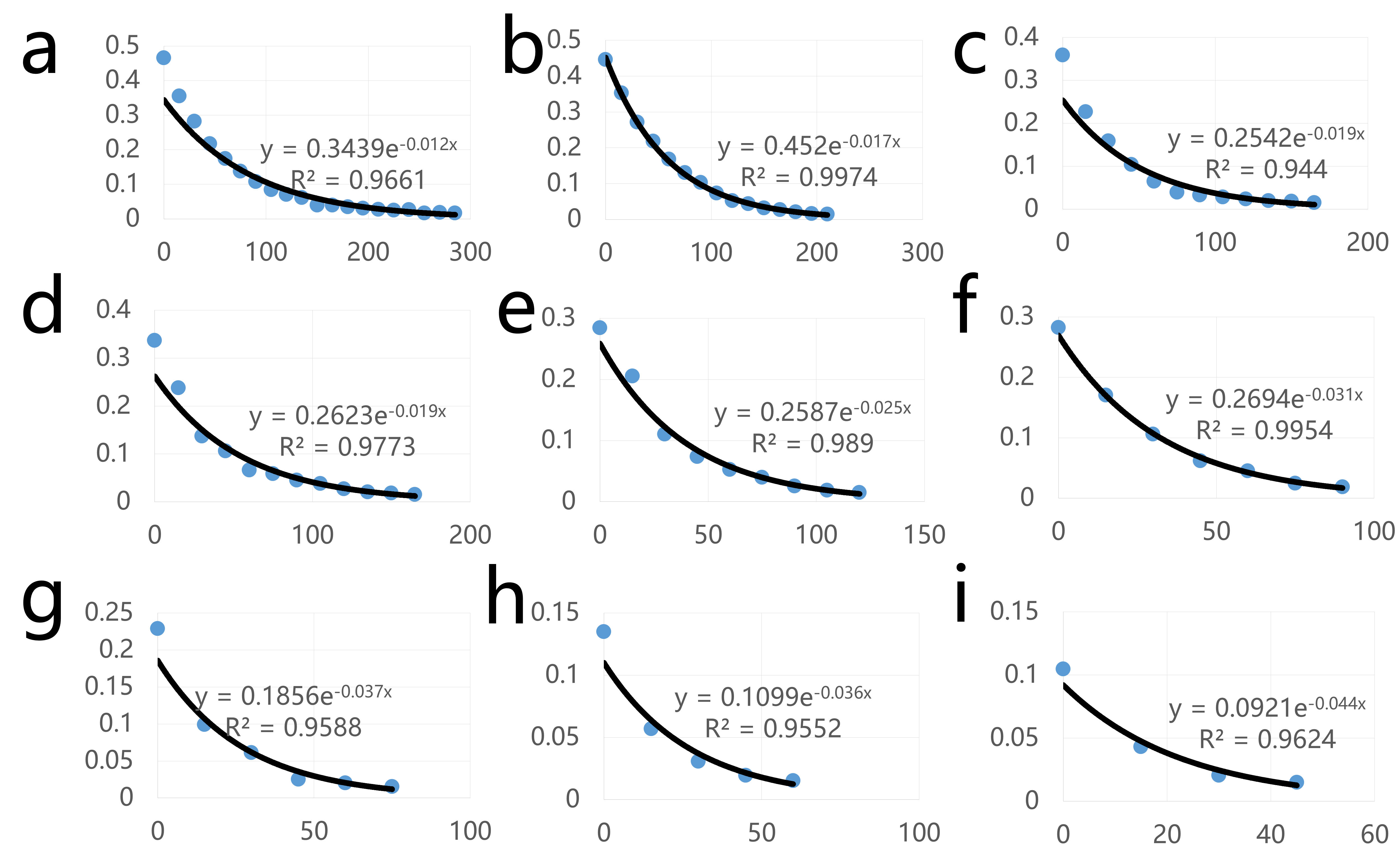

Supplement: S2 Fig — Levitation heights of the upper (blue) and lower (orange) limits of confinement of a sample of polystyrene microspheres over time. Time 0 corresponds to when the sample was inserted into the magnetic field and data was collected until the sample reached equilibrium. Nine different gadolinium concentrations are shown in a-i. Each time point is an average over six trials with error bars representing the standard deviation. (TIF) [file pone.0134400.s003.tif]

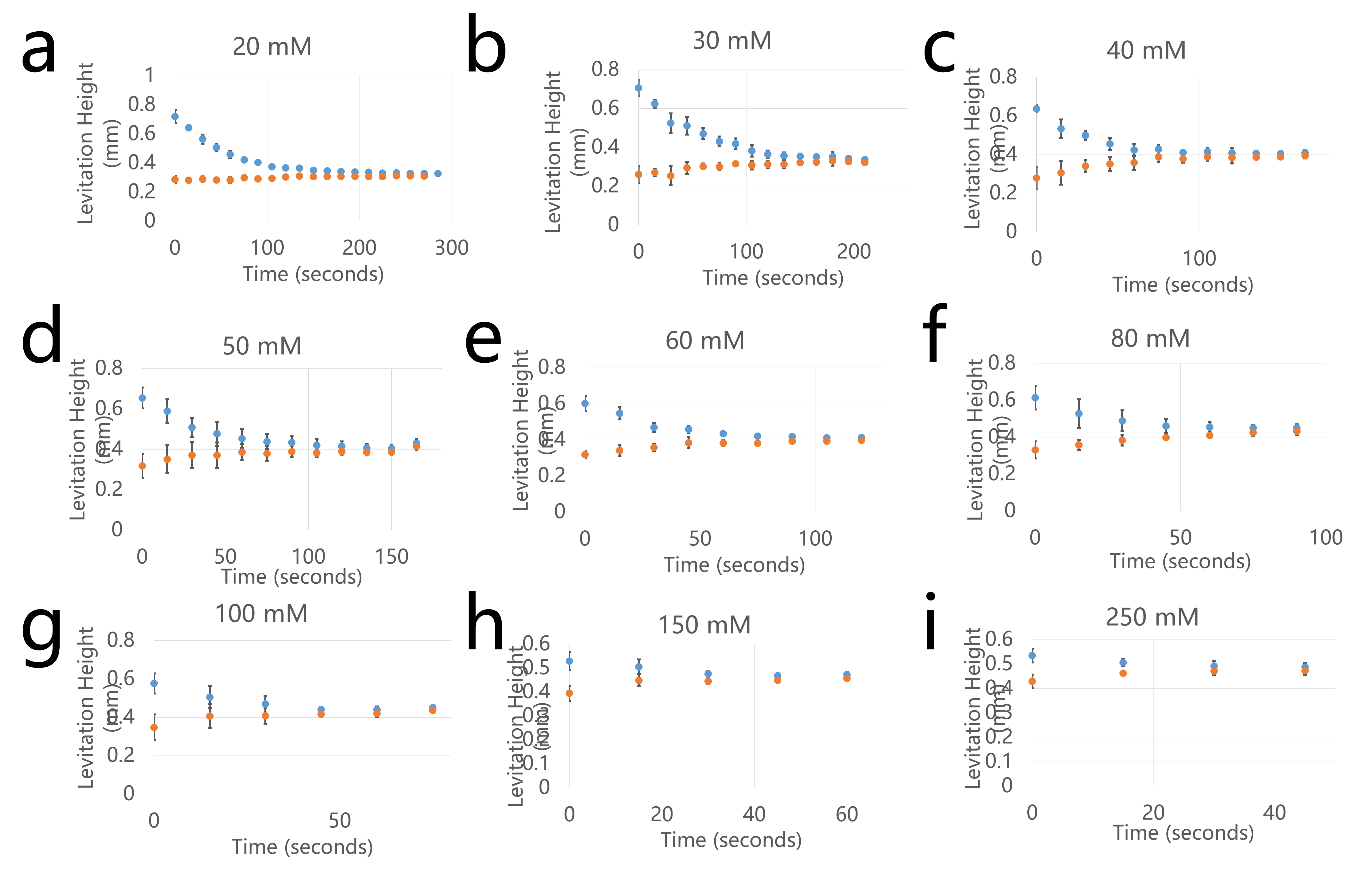

Supplement: S3 Fig — Width between the upper and lower limits of confinement of a sample of polystyrene microspheres over time. Values are calculated as the difference between the upper and lower data points shown in S2 Fig Nine concentrations of gadolinium paramagnetic solution were tested. An exponential decay equation was used to approximate the data with R2 greater than 0.94 in all cases. (TIF) [file pone.0134400.s004.tif]

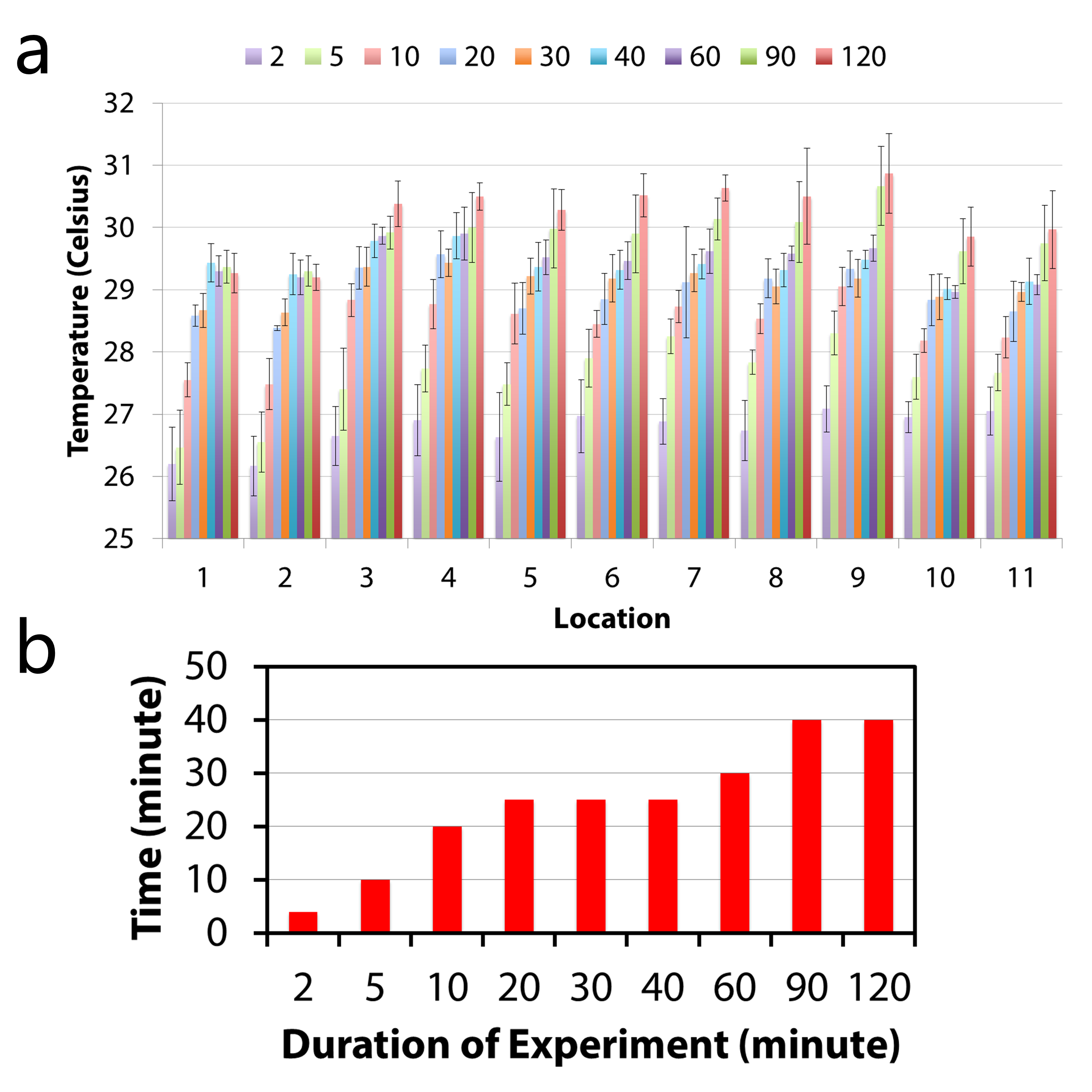

Supplement: S4 Fig — After the smart-phone camera application was turned on, the temperature was measured at 11 locations on the back surface of smart-phone (Fig 4A) for time points: 2, 5, 10, 20, 30, 40, 60, 90, and 120 minutes (b). At the battery side, the temperature was measured at 5 locations (four on the edge of battery, and one at the middle of the battery). At the camera side, temperatures of six locations were measured, including the location corresponding to the end of capillary tube (8) and the temperature on the camera (9). After each measurement, the smart-phone application was turned off for cooling until the temperature of smart-phone decrease to room temperature. Six repeats for each of eleven locations at the back surface of the smart-phone. (c) Cooling times required between each trial for different thermal exposure times. For instance, for 90 minute experiments durations, the smart-phone was kept off for 40 minutes prior to the start of a new trial. (TIF) [file pone.0134400.s005.tif]
